# New fossil of *Gaoloufangchaeta* advances the origin of Errantia (Annelida) to the early Cambrian

**DOI:** 10.1098/rsos.231580

**Published:** 2024-04-10

**Authors:** Xiaoyu Yang, M. Teresa Aguado, Conrad Helm, Zhiqian Zhang, Christoph Bleidorn

**Affiliations:** ^1^ Yunnan Key Laboratory for Palaeobiology, Institute of Palaeontology, Yunnan University, Kunming 650500, People's Republic of China; ^2^ Animal Evolution and Biodiversity, Georg-August-Universität Göttingen, Göttingen 37073, Germany; ^3^ School of Fine Arts, Yunnan Normal University, Kunming 650500, People's Republic of China

**Keywords:** Guanshan biota, Errantia, crown annelids, palaeoecology, early Cambrian

## Abstract

Molecular clock estimates suggest the origin of Annelida dates back to the Ediacaran period, which is in discordance with the first appearance of this taxon in the early Cambrian, as evidenced by the fossil records of stem-group and basally branching crown-group annelids. Using new material from the early Cambrian Guanshan biota (Cambrian Series 2, Stage 4), we re-interpret *Gaoloufangchaeta bifurcus* Zhao, Li & Selden, 2023, as the earliest known errantian annelid. *Gaoloufangchaeta* has a prominent anterior end bearing three pairs of putatively sensory appendages and a pair of anterior eyes; a muscular eversible pharynx with papillae is identified. The presence of enlarged parapodia with acicula-like structures and long capillary chaetae suggests a pelagic lifestyle for this taxon. Our phylogenetic analyses recover *Gaoloufangchaeta* within the Phyllodocida (Pleistoannelida, Errantia), extending the origin of Errantia back to the early Cambrian. Our data are in line with the hypothesis that Annelida diverged before the Cambrian and indicate both morphological and ecological diversification of annelids in the early Cambrian.

## 1. Introduction

Annelida is a phylum of segmented worms with around 20 000 described recent species showing a great diversity of morphology and lifestyles [1]. According to recent phylogenetic analyses, annelids comprise a major clade, Pleistoannelida, as well as a paraphyletic grade of basally branching taxa, including Palaeoannelida (Magelonidae + Oweniidae), Chaetopteriformia, Amphinomida and Sipuncula [2]. Pleistoannelida is divided into two groups, Errantia and Sedentaria, which are mainly named after their life habits and contain the highest annelid diversity [3]. Molecular clock estimates suggest that Annelida originated in the Ediacaran period, but the origin of the annelid crown group was probably within the early Cambrian [4,5], in accordance with the ‘Cambrian Explosion’. Thus, the discovery of crown annelids from this interval is crucial to our knowledge about the origin and early diversification of crown-group annelids and will help us to understand the emergence and evolution of the diverse morphologies in this group [6].

Reliable fossils of Cambrian annelids are relatively scarce owing to their soft bodies which are prone to decay. All representatives are documented from the early and middle Cambrian exceptionally preserved fossil Lagerstätten such as the Canadian Burgess Shale [7–10], the Sirius Passet fauna of Greenland [11,12], the Chengjiang biota [13], Hongjingshao Formation [14] and Guanshan biota [15,16] of China. The majority of Cambrian annelids show similarities in morphology, such as paired prostomial palps and biramous parapodia bearing simple capillary chaetae, to recent forms, but according to cladistic analyses, they fall outside of the annelid crown group [8,14,17].

The earliest robust evidence of crown annelids comes from the records of early Cambrian representatives of Palaeoannelida [14] and Sipuncula [18], both representing basally branching groups of Annelida. *Iotuba* from the early Cambrian Chengjiang Biota which has recently been interpreted as a taxon within Sedentaria [19] suspiciously lacks typical characteristics of annelids, such as segments, palps and chaetae, leaving its annelid affinity highly questionable. There has been no fossil record of the diversified Errantia until the Cambrian–Ordovician transition, as evidenced by the appearance of scolecodonts, which are interpreted as the jaws of Eunicida, Errantia [20]. The oldest unambiguous body fossils of Phyllodocida, Errantia, are plumulitid machaeridians from the early Ordovician of Morocco [21,22].

Herein and based on new material from the early Cambrian Guanshan biota (Cambrian Series 2, Stage 4), we re-interpret *Gaoloufangchaeta* Zhao, Li & Selden, 2023, originally described as epibenthic, as a pelagic member of Errantia. A detailed investigation of morphological characteristics and subsequent phylogenetic analyses strongly support an earlier origin and diversification of annelid crown groups than previously assumed.

## 2. Material and methods

### 2.1. Material

The fossil specimen YKLP 12465 described here was collected from the Wulongqing Formation (Cambrian Stage 4, *ca* 514–509 Myr) at the Gaoloufang section, Kunming, Yunnan Province, southwestern China, and is housed at the Yunnan Key Laboratory for Palaeobiology (YKLP), Yunnan University.

### 2.2. Fossil imaging

**Figure 1.**
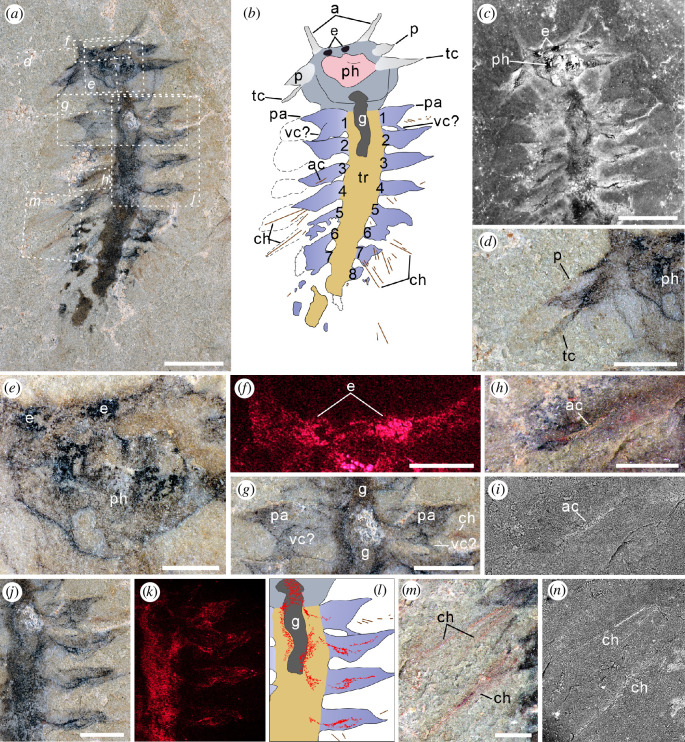
*Gaoloufangchaeta* from the early Cambrian Guanshan biota, part of YKLP 12465. (*a,b*) View of the entire fossil, anterior is up: (*a*) light microscopic imaging and (*b*) interpretative line drawing. Colours indicate the head (grey-blue), eyes (black), head appendages (light grey), pharynx (pink), trunk (yellow), gut (dark grey) and parapodia (blue) with chaetae (brown). Dashed lines around the parapodia indicate chaetal areas. Numbers denote chaetigers. (*c*) Fluorescent microscopic imaging of the head and anterior segments. (*d*) A close-up of the boxed area in (*a*), showing the detail of the palp and tentacular cirrus on the right side. No associated chaetae are observed. (*e*) A close-up of the boxed area in (*a*), showing the detail of the eyes and pharynx. (*f*) EDS mapping of carbon about eyes. (*g*) A close-up of the boxed area in (*a*), showing the detail of chaetiger 1 with parapodia. (*h*) A close-up of the boxed area in (*a*), showing the detail of the aciculum. (*i*) A BSE image of (*h*). (*j–l*) Detail of the branched tissue of unknown origin: (*j*) a close-up of the boxed area in (*a*); (*k*) EDS mapping of carbon; and (*l*) interpretative line drawing, with the tissue in red. (*m*) A close-up of the boxed area in (*a*), showing the detail of the chaetae. (*n*) A BSE image of (*m*). a, antennae; ac, aciculum; ch, chaetae; e, eyes; g, gut; p, palps; pa, parapodia; ph, pharynx; tc, tentacular cirri; tr, trunk; vc, ventral cirrus. Scale bars: (*a–c*) 2 mm, (*d,g*,*j–l*) 1 mm, (*e,f,h,m,n*) 500 μm.

**Figure 2.**
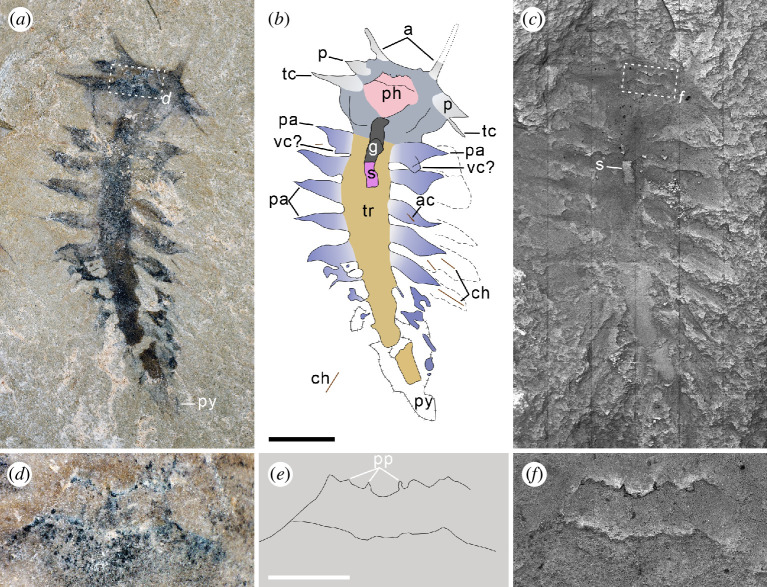
*Gaoloufangchaeta* from the early Cambrian Guanshan biota, the counterpart of YKLP 12465, anterior is up in all images. (*a–c*) Whole view: (*a*) light microscopic imaging; (*b*) interpretative line drawing. Colours indicate the head (grey-blue), head appendages (light grey), pharynx (pink), trunk (yellow), gut (dark grey) filled with sediment (violet) and parapodia (blue) with chaetae (brown). Dashed lines around the parapodia indicate chaetal areas; and (*c*) BSE imaging. (*d*) A close-up of the boxed area in (*a*), showing the detail of the pharynx. (*e*) An interpretative line drawing of the pharynx with papillae. (*f*) A close-up of the boxed area in (*c*), showing the detail of the pharynx. a, antennae; ac, aciculum; ch, chaetae; g, gut; p, palps; pa, parapodia; ph, pharynx; pp, papillae; py, pygidium; s, sediment; tc, tentacular cirri; tr, trunk; vc, ventral cirrus. Scale bars: (*a–c*) 2 mm, (*d–f*), 500 μm.

The specimen was prepared manually with fine needles under a Nikon SMZ800 stereomicroscope and then photographed using a Keyence VHX 6000 stereomicroscope and a Leica DFC7000 T monochrome digital camera attached to a Leica M205 FA fluorescence stereomicroscope (figure 1*c*). Backscattered electron (BSE) image capture and energy-dispersive x-ray spectroscopy (EDS) analysis were performed using an FEI Quanta 650 scanning electron microscope (SEM) under low vacuum and high accelerating voltage (30 kV) (figure 1*f,k,i,n*) and a JEOL JSM-IT500 SEM under low vacuum with a voltage of 15 kV (figure 2*c,g*; electronic supplementary material, figure S1). Photographs were processed in Adobe Photoshop and CorelDRAW SE 2021.

### 2.3. Phylogenetic analysis

Phylogenetic analyses were conducted based on the published morphological character matrix of Chen *et al*. [14]. Most taxa belonging to Brachiopoda and Mollusca and their related characters were removed; Machaeridians preserved without parapodia and chaetae were removed; *Gaoloufangchaeta*, *Ursactis* [10] and three extant pelagic taxa (Alciopidae, Lopadorrhynchidae and Tomopteridae) [23,24] were added; some of the character codings were modified in order to maintain consistency in character coding (see the electronic supplementary material for details). The matrix contains 90 taxa and 242 characters (see the electronic supplementary material). Bayesian analyses with and without topological constraints were performed using MrBayes 3.2.7, following the published MrBayes block of Chen *et al*. [14], with the Mki model, gamma distribution for relative rates [25] and default priors for all parameters. *Myzostoma* was excluded owing to its uncertain affinity with Errantia [2]. One hundred million generations with 25% burn-in were generated, and the analysis was stopped once the average deviation of split frequencies fell below 0.01. Convergence was assessed by an estimated sample size larger than 200 and a potential scale reduction factor equal to 1.00 for each parameter. Topological constraints contained results from widely accepted phylogenomic studies of annelids (see the electronic supplementary material; [2]).

## 3. Systematic palaeontology

Phylum Annelida Lamarck, 1809 [26]

Clade Pleistoannelida Struck, 2011 [3]

Subclass Errantia Audouin & Milne Edwards, 1832 [27]

Clade Aciculata Rouse & Fauchald, 1997 [28]

Order Phyllodocida Dales, 1962 [29]

*Gaoloufangchaeta bifurcus* Zhao, Li & Selden, 2023 [16]

### 3.1. Holotype

An incomplete specimen, RCP-ZJ-0001 (Research Center of Paleobiology, Yuxi Normal University) (Figures 2–4 in [16]).

**Figure 3.**
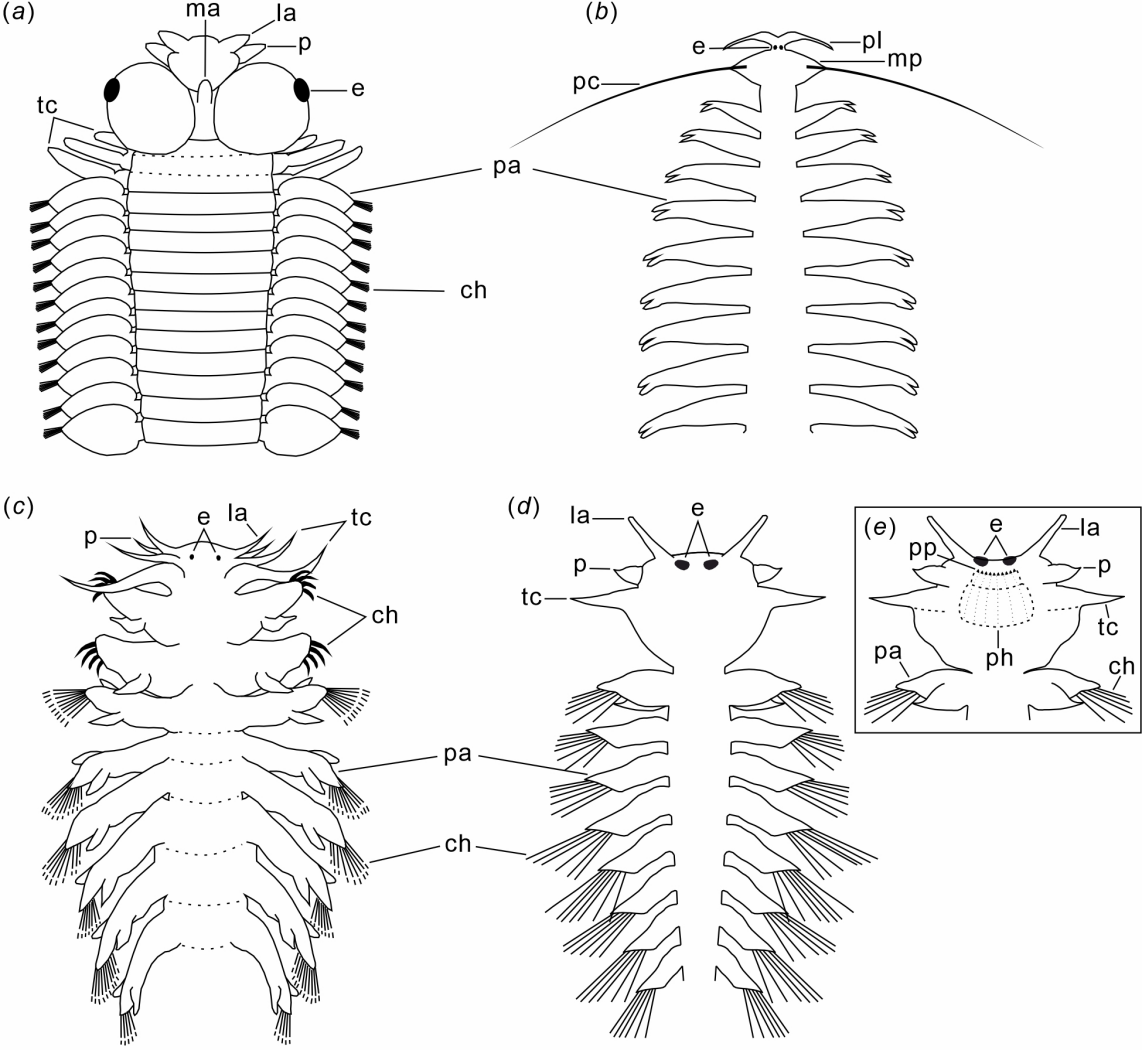
Comparative diagrams showing the morphologies of the anterior parts of extant and Cambrian pelagic annelids. (*a*) *Rhynchonerella angelini* (Alciopidae), dorsal view (modified from [23], text-fig. 13; [30], figs 13–38B). (*b*) *Tomopteris pacifica* (Tomopteridae), dorsal view (modified from [23], text-fig. 6). (*c*) *Lopadorhynchus krohnii* (Lopadorhynchidae), dorsal view (modified from [23], text-fig. 21). (*d,e*) *Gaoloufangchaeta bifurcus*: (*d*) dorsal view (from the holotype in [16] and the fossil specimen here); and (*e*) ventral view of the head bending a little ventrally with the possible everted pharynx. ch, chaetae; e, eyes; la, lateral antennae; ma, median antenna; mp, modified parapodia; p, palps; pa, parapodia; pc; parapodial cirri; ph, pharynx; pl, palp-like appendages; pp, papillae; tc, tentacular cirri.

**Figure 4.**
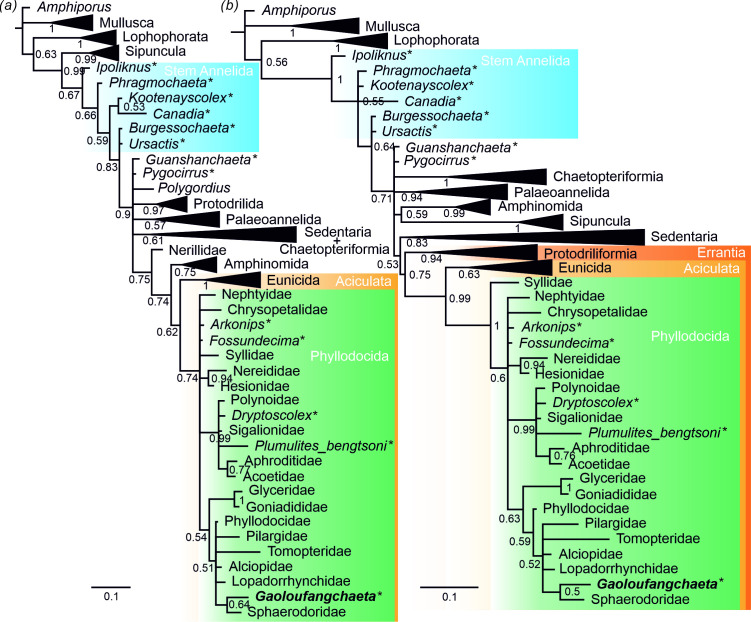
Phylogenetic position of *Gaoloufangchaeta* revealed by Bayesian analysis. (*a*) Topology of unconstrained analysis. (*b*) Topology of analysis with constraints. The nodes are labelled with posterior probabilities. The scale bar represents the expected changes per site. Fossil taxa are indicated by asterisks.

### 3.2. Other material

YKLP 12465, part and counterpart (figures 1 and 2; electronic supplementary material, figure S1).

### 3.3. Occurrence

Wulongqing Formation (Cambrian Stage 4); Gaoloufang section, Kunming, Yunnan Province, southwestern China.

### 3.4. Revised diagnosis

Polychaete having a muscular eversible pharynx with papillae. The head relatively big, with three pairs of appendages and a pair of anterior eyes. The trunk tapering posteriorly, bearing at least eight chaetigers with uniramous parapodia. Each parapodium enlarged, bearing one acicula-like structure and elongated capillary chaetae in bundles.

### 3.5. Description

The specimen appears dark in general, with the dark tissues being identified by EDS as carbonaceous remains and iron oxides (figure 1; electronic supplementary material, figure S1). The specimen is almost complete and has a prominent prostomium and at least eight chaetigers (figures 1*a*–*c* and 2*a–c*). The preserved length of the specimen is about 11.2 mm, of which the head occupies about 21%. The head achieves its widest (*ca* 3.4 mm) in the mid and tapers anteriorly and posteriorly. A pair of elongated appendages extend from the anterior margin of the head (figures 1*a*–*c* and 2*a–c*). The distance between the two appendages is about 1.2 mm; each has an approximately 0.4 mm wide base and tapers distally with a length of more than 1 mm (figures 1*a*–*c* and 2*a–c*). A pair of oval structures (0.2 mm in width and 0.3 mm in length) preserved as dark carbonaceous film (figure 1*a*–*e*) is located between appendages and shortly behind the anterior margin, indicating that they are remnants of eyes. Behind these eyes a two-ringed structure with a U-shaped posterior margin is situated and bears several tiny spine-like papillae (*ca* 50 μm in width and 55 μm in length) (figures 1*a*–*c,e* and 2). This structure is interpreted here as pharynx with distal papillae. Although prominent papillae are visible, there is no evidence of sclerotized jaws within the structure. The presence of a partly everted pharynx suggests that the specimen is preserved in ventral view. The pharynx is flanked by two pairs of appendages with wide basal parts (*ca* 0.4 mm) and pointed tips (figures 1*a*–*d* and 2*a–c*). No associated chaetae are observed (figure 1*d*). The posterior pair of appendages (*ca* 1.6 mm in length) is longer and located more ventrally than the anterior appendages (*ca* 0.8 mm in length); their bases are adjacent to the pharynx (figures 1*a*–*c* and 2*a,b*). Their differences in shape suggest that they represent different head appendages like palps or tentacular cirri. Several dark lines enriched in carbon are located in the posterior part of the head (figures 1*a*,*b* and 2*a,b*), possibly representing the thickened epidermis owing to compaction. The constriction of the posterior part of the head that is also present in the holotype [16] is more morphological than taphonomic, putatively indicating the boundary between the head and the trunk. As there is no evidence of a ring-shaped structure separating the prostomium from the remaining segments, the peristomium may be just confined to distinct lips as exhibited by many modern errant polychaetes [31].

A dark tube partly three-dimensionally preserved, extending from the pharynx and running along the anterior trunk (figures 1*a*–*c*,*g,j*–*m* and 2*a–c*) is interpreted as the gut. The gut is preserved as carbonaceous film (figure 1*k*). The trunk is widest in chaetiger 3 (*ca* 1.5 mm) and then tapers posteriorly (figures 1*a*–*c* and 2*a–c*). Each chaetiger bears a pair of lobe-shaped parapodia extending laterally. The parapodia appear uniramous and similar in size (*ca* 1.5–1.7 mm) (figures 1*a*–*c* and 2*a–c*); those of chaetiger 4 extend up to 1.75 times the width of the corresponding chaetiger (figures 1*a*–*c* and 2*a–c*). Posterolateral to each parapodium of chaetiger 1–5 on the right side occurs an area that is slightly different in colour from the matrix (figures 1*a*–*c* and 2*a–c*); within the fourth and fifth areas, two or three simple, elongated chaetae appearing to be in bundles (figures 1*a*,*b*,*m,n* and 2*a,b*) can be observed. These chaetae are similar to those of the parapodium on the left side of chaetiger 1 and have a maximum length and width of 1.5 mm and 30 μm, respectively (figure 1*m,n*). The actual number of chaetae in each bundle is hard to estimate. The rod-like structure (figure 1*h,i*) inside the parapodium of chaetiger 3, which is shorter and wider than the chaetae (*ca* 0.6 mm long and 45 μm wide), is interpreted as being an acicula-like structure. Both chaetae and the putative aciculum are preserved as iron oxides in framboids (figure 1*i,n*; electronic supplementary material, figure S1f), indicating their original composition of pyrites. A lobe connected to the base of each parapodium on chaetiger 1 is preserved overlying the parapodium as evidenced by a clear anterior margin (figure 1*a,g*) and is relatively smaller in size. No chaetae or aciculae associated with it are observed. It might represent a structure comparable to the ventral cirrus or ventral parapodial ligule as in phyllodocidan polychaetes [32].

Carbonaceous striped traces in the anterior part of the trunk are preserved surrounding the gut (figure 1*j*–*l*). The left stripe is observed to have evenly spaced lateral extensions entering the first four parapodia on the left side at a median position (figure 1*j*–*l*). Each extension forks and meets again, resulting in a distinctive medial gap (figure 1*j*–*l*), showing distinction from the margin of the corresponding parapodium. These structures are so far unidentified and probably represent neuronal or muscular tissue.

The specimen shows no evidence of nuchal organs, dorsal cirri or branchia. The features of the posterior trunk and pygidium are unclear owing to poor preservation.

### 3.6. Remarks

This specimen is consistent with the holotype of *Gaoloufangchaeta* [16] in overall morphology: a pair of anterolateral appendages, a pair of eyes, an everted pharynx and enlarged parapodia. The second and third pairs of appendages on the head are most likely the putatively sensory head appendages rather than parapodia as interpreted in the holotype [16]. In such a case, parapodia occur only on the trunk of the holotype and the number is at least five pairs. Moreover, the pygidium observed in the holotype [16] seems to contain two lobes which are preserved in different layers and are similar in morphology and size to the anterior paired parapodia. Given that the specimen described here has at least eight pairs of parapodia, it seems reasonable to interpret the ‘pygidium’ of the holotype as bearing parapodia. The structures interpreted as chaetae in the holotype [16] are inside the parapodia and are much thicker than those of the specimen described here, indicating they are aciculae.

The observation of an everted pharynx suggests the holotype is preserved in ventral view as well. Thus, the interpretation of possible nuchal organs in the holotype seems unlikely as these epidermal sensory structures of recent annelids are usually situated dorsally or dorsolaterally at the posterior end of the prostomium [33]. According to their close association with the mouth (pharynx), the described structures most likely represent the margins of the everted pharynx.

The evidence of eyes with a position slightly behind the anterior margin of the head and compaction lines on the posterior of the head suggests that the head may have been bent a little ventrally when being buried, and the eyes were originally at the anterior part on the dorsal side of the prostomium.

## 4. Discussion

### 4.1. Morphology

The terms antennae, palps and tentacular cirri are used to describe various, mainly sensory, appendages on the head of annelids [34]. Antennae are usually located dorsally or at the anterior margin of the prostomium, while palps tend to be located ventrally or laterally and are usually associated with the mouth opening, functioning as a feeding or sensory organ [24,35]. In many annelids (e.g. Phyllodocida), one or more anterior segments that become cephalized and differ in size and shape from the following only bear paired parapodial cirri called tentacular cirri [28,36]. Therefore, the first pair of elongated appendages which are located at the margin of the head of our specimen most likely represent antennae; the second pair which are conical situated ventrolaterally and close to the pharynx are probably palps; the third pair, slender than the palps, may represent the tentacular cirri of the cephalized first segment. Unlike the feeding palps of other Cambrian taxa (e.g. *Canadia* [9] and *Dannychaeta* [14]) and some recent annelids (e.g. Magelonidae, [37]) which are elongated and grooved, those of *Gaoloufangchaeta* are smooth and relatively short, thereby probably representing sensory palps as exhibited by the majority of Errantia [38]. The tentacular cirri in modern annelids typically contain both dorsal and ventral pairs, representing dorsal parapodial cirri and ventral parapodial cirri, respectively. However, only one pair of tentacular cirri on one segment is also present in certain errant annelids such as Tomopteridae that have one pair of long tentacular cirri on segment 2 [23,24]. The single pair of tentacular cirri in *Gaoloufangchaeta* is on the ventral surface of the head, most likely representing the ventral cirri on the first segment.

The presence of a differentiated head, and uniramous, enlarged parapodia distinguishes *Gaoloufangchaeta* from other Cambrian annelids. Parapodia are prominent and enlarged as in modern errant annelids [38], and each is inferred to bear one aciculum. An alternative interpretation of the parapodia on chaetiger 1 is that they are biramous based on two lobes. However, the smaller size and no observation of associate chaetae and aciculae of the ventral lobe make this possibility less reasonable. Uniramous parapodia present in *Gaoloufangchaeta* are not common in recent annelids. Members of Eunicida have reduced notopodia with no notochaetae, but the presence of dorsal cirri with embedded notoaciculae proves that the parapodia are biramous [39]. The anterior body region of Chaetopteridae bears uniramous parapodia [40], showing a certain similarity with *Gaoloufangchaeta*. However, chaetopterids have an indistinctive head, a pair of palps, eyespots, noneversible pharynx and cutting chaetae in chaetiger 4 in the anterior region [40], which are different from the distinct head, three pairs of head appendages, big eyes, eversible pharynx and simple elongated chaetae in *Gaoloufangchaeta*. Uniramous parapodia also occur in certain specialized polychaetes, such as pelagic Alciopidae ([23]; figure 3*a*) and Lopadorrhynchidae ([23]; figure 3*c*), as well as in commensal parasitic Myzostomida [41].

*Gaoloufangchaeta* is observed to have only ventral cirri on chaetiger 1. The absence of dorsal and/or ventral cirri on the trunk—although unusual among errant annelids—occurs in some taxa, such as Myzostomida and Sphaerodoridae without dorsal cirri [41,42] and Tomopteridae lacking both dorsal and ventral cirri [23].

The eyes of *Gaoloufangchaeta* were previously considered to be bicellular [16]. However, they are most likely multicellular based on their relatively large size, as in many extant annelids, such as Amphinomidae, Aciculata (Phyllodocida + Eunicida) and some sedentarians (e.g. Flabelligeridae and Orbiniidae) [43]. The discovery of *Gaoloufangchaeta* provides the earliest evidence of annelid eyes [16] and indicates that multicellular eyes of Annelida have already evolved in the early Cambrian.

Internal carbonaceous striped structures may represent neural tissues or muscle tissues. The former is evidenced by similarity in shape to the ventral nerve cord and parapodial nerves as observed in *Canadia* [9] and extant *Neanthes* [44], and the organic preservation of nerve tissues which is quite common in Cambrian Burgess Shale-type fossils [45]. However, this possibility is difficult to reconcile with their large sizes, the lack of fine neurite branches and the difficulty of the fossilization of epidermal neuronal tissues. Their position relative to the gut and relatively large sizes support the latter. However, fossilized muscle tissues are predominately phosphatized as in the Cretaceous annelid *Rollinschaeta* [46]. The identification of these striped structures relies on more fossils of *Gaoloufangchaeta*.

### 4.2. Palaeoecology

*Gaoloufangchaeta* is inferred to represent a pelagic taxon based on morphological evidence. The presence of eyes and the visibility of more than one pair of anterior putatively sensory appendages (antennae and palps) suggest that *Gaoloufangchaeta* exhibits a well-developed sensory system. Furthermore, the presence of enlarged parapodia with internal aciculae and long capillary chaetae, and possible well-developed bundles of longitudinal as well as parapodial muscles indicate its potential of having a rapid and powerful movement and suggest they are suitable for undulating movement.

There are just a few extant annelid groups with a pelagic lifestyle, and each has unique morphological adaptations [23,47,48]. Most of them (e.g. Alciopidae, Lopadorrhynchidae and Tomopteridae) belong to Phyllodocida. Alciopidae, Lopadorrhynchidae and Tomopteridae have evolved to have transparent bodies. Large eyes and eversible proboscis with horns and papillae have developed in Alciopidae. Lopadorrhynchidae exhibits flattened compound chaetae, whereas Tomopteridae has paddle-shaped parapodia without chaetae for their undulate swimming behaviour. The combination of morphological characters, such as various sense organs, similar segments as well as well-developed parapodia and chaetae, suggests that *Gaoloufangchaeta* has a phenotype that is comparable to that of these three recent holopelagic taxa (figure 3).

The presence of a papillated and possibly muscular pharynx as known for Alciopidae indicates that *Gaoloufangchaeta* was probably an active feeder and potential predator, although the gut is observed to be partly three-dimensionally preserved. These gut contents have been interpreted either as sediment infillings as evidence of deposit feeding or as clay minerals owing to the weathering of gut phosphatization [49]. Even if the former is followed, considering the specimen is preserved nearly completely in the fine-grained mudstone and the sediment appears at the anterior part of the gut, the sediment may just have entered the gut during burial.

Most Cambrian annelids such as *Phragmochaeta* [11], *Canadia* [9], *Kootenayscolex* [8] or *Ursactis* [10] have been interpreted as epibenthic forms owing to the combined evidence of morphology (such as prominent but not well-developed parapodia and no eyes and aciculae) and taphomomy (such as possible sediment infills through the whole gut or aggregation preservation); some are inferred to have an endobenthic lifestyle such as *Peronochaeta* [7] or *Dannychaeta* [14]. Additionally, a commensalistic relationship with tubicolous enteropneusts is described for an unnamed annelid from the Burgess Shale [50], leaving the *Gaoloufangchaeta* as the earliest known annelid with a potentially pelagic lifestyle.

### 4.3. Phylogeny

The results of our phylogenetic analyses (figure 4; electronic supplementary material, figure S2) are mostly consistent with those of previous studies [10,14,51], revealing most Cambrian polychaetes as stem-group annelids, and *Guanshanchaeta* and *Pygocirrus* in a polytomy with other crown-group annelids; Sipuncula was placed outside the Annelida (figure 4; electronic supplementary material, figure S2a), an artefact probably caused by its loss of key characters such as segmentation [51,52]. *Gaoloufangchaeta* was placed deeply within crown-group Annelida, and more specifically, within Phyllodocida (Aciculata, Errantia) in our phylogenetic analyses, whether topological constraints based on phylogenomic analyses are applied or not (figure 4; electronic supplementary material, figure S2). Especially, the presence of paired antennae, palps and tentacular cirri, enlarged parapodia and a muscular pharynx with papillae unite *Gaoloufangchaeta* with Phyllodocida.

The placement of Cambrian *Guanshanchaeta* in a polytomy with crown-group Annelida in our analyses and previous studies [10,51] indicates that the presence of aciculae can be interpreted as apomorphic for crown-group Annelida although ancestral reconstructions revealed aciculae as elements of the annelid ground pattern [53]. Aciculata, which was originally named based on the presence of aciculae [28], now contains only two clades, Eunicida and Phyllodocida [54]. Characteristically, extant members of Phyllodocida bear sensory ventral palps, an anterior enlarged cirri, axial muscular pharynx and compound chaetae [55]. Some of these features are absent in *Gaoloufangchaeta,* which may indicate character losses in this fossil.

The placement of *Guanshanchaeta* in Phyllodocida importantly extends the fossil records of Phyllodocida and Errantia back to the early Cambrian during which representatives of other clades of annelids also appeared [14,18], thus providing further indirect support to the Ediacaran hypothesis for the origin of Annelida [4,5]. Even though the annelid affinity of *Iotuba* [19] is highly questionable, we would expect to find fossils representing Sedentaria from the exceptionally preserved biotas from the early Cambrian. Pelagic *Gaoloufangchaeta*, together with previously reported epibenthic stem-group annelids [7–10] and endobenthic palaeoannelids [14] from the early Cambrian, reveal the diversification of not only the morphology but also the lifestyle of annelids in the ‘Cambrian Explosion’.

## Data Availability

All data are available in the main text or within the electronic supplementary material (figures S1–S2 and other information about phylogenetic analyses) [56].
